# Potential of Caffeic Acid and 10-Dehydrogingerdione as Lipid Regulators Relevant to Their Inhibitory Effect on miR-122 and ATP Citrate Lyase Activity in Diabetic Hyperlipidemic Rats

**DOI:** 10.3390/biomedicines11030726

**Published:** 2023-02-28

**Authors:** Mohamed M. Elseweidy, Alaa S. Elawady, Mohammed S. Sobh, Abdulmohsen H. Alqhtani, Naif A. Al-Gabri, Gehad M. Elnagar

**Affiliations:** 1 Department of Biochemistry, Faculty of Pharmacy, Zagazig University, Zagazig 44519, Egypt; 2 Pathology Department, Faculty of Veterinary Medicine, Zagazig University, Zagazig 44519, Egypt; 3 Department of Animal Production, College of Food and Agriculture Sciences, King Saud University, Riyadh 11362, Saudi Arabia; 4 Department of Pathology, Faculty of Veterinary Medicine, Thamar University, Dharma 124401, Yemen; 5 Laboratory of Salam Veterinary Group, Burydha 51911, Saudi Arabia

**Keywords:** caffeic acid, 10-DHG, miR-122, CCT-diet, ACLY, p-AMPK

## Abstract

The present study aimed to illustrate the hypolipemic effect of 10-Dehydrogengardione (10-DHG) or caffeic acid (CA) with reference to the role of microRNA-122 (miR-122) and ATP citrate lyase (ACLY) activity. Diabetic hyperlipidemia was induced in rats, and then randomly classified into three groups. The first one received only a CCT-diet for 6 weeks and was referred to as the positive control. The other two groups received 10-DHG (10 mg/kg/day) or CA (50 mg/kg/day), orally for 6 weeks along with a CCT-diet. Another group of normal rats was included, received a normal diet, and was referred to as the negative control. Either 10-DHG or CA significantly decreased MiR-122 expression and appeared more remarkable in the CA group by 15.5%. The 10-DHG greatly enhanced phosphorylated form of AMP activated protein kinase (p-AMPK) activity, more than CA by 1.18-fold, while the latter exerted more inhibitory effect on ACLY, and fatty acid synthase (FAS) activities compared with 10-DHG (*p* < 0.05). Both drugs significantly decreased hydroxy methyl glutaryl coenzyme A (HMG-COA) reductase activity, which appeared more remarkable in 10-DHG, and significantly decreased triglyceride (TG), total cholesterol (TC), and low-density lipoprotein cholesterol (LDL-C) along with a high density lipoprotein cholesterol (HDL-C) increase. The 10-DHG ameliorated the hepatic tissue lesions greatly, more than CA. The 10-DHG or CA significantly inhibited MiR-122, hepatic FAS, and ACLY levels along with p-AMPK activation. This subsequently led to reduced plasma TG, cholesterol levels, and blood glucose improvement and, indeed, may explain their mechanisms as hypolipemic agents.

## 1. Introduction

MicroRNAs (miRNAs) are a well-known class of short non-coding RNAs that controls the expression of particular target genes through binding to complementary regions on mRNA, cleaving or blocking the translation of target MRNA. miRNAs can serve in additional significant regulatory roles in a wide range of biological processes, and more than 60% of human genes are modulated by miRNAs [1]. The latter control various metabolic pathways including insulin secretion and glucose and lipid metabolism [2], and its downregulation exerts a certain role in the development of chronic disorders such as obesity, NAFLD, type 2 diabetes, and cardiovascular diseases [3].

MiR-122 is a unique liver-specific one that constitutes nearly 70% of the hepatic miRNAs [4], and several genes that control TG and fatty acid synthesis are regulated by miR-122, including FAS and ACLY [5]. miR-122 inhibition, on the other hand, can remarkably decrease certain important genes involved in cholesterol biosynthesis such as HMG-CoA reductase [6].

Lipid metabolism is controlled also by many lipogenic proteins, including FAS, which is involved in lipolysis and lipogenesis. The latter includes FAS participation in the catalysis of acetyl-CoA conversion to malonyl-CoA; FAS in excess can impair lipid metabolism, leading to significant fat accumulation and many metabolic disorders such as NAFLD, type 2 diabetes, cardiovascular disease, and obesity [7].

ACLY is an important enzyme involved in the synthesis of cholesterol and fatty acids, as well as in the catabolism of other nutrients, abundantly expressed in the liver, adipose, and other lipogenic tissues in mammals. ACLY catalyzes the conversion of citrate into acetyl-CoA and oxaloacetate in the presence of ATP and CoA [8], and deregulation of either the activity or the liver protein expression may be connected to NAFLD, hyperlipidemia, or type 2 diabetes mellitus [9].

The main metabolic sensor, AMPK, is an important regulatory enzyme that enhances ATP-generating pathways such as fatty acid oxidation along with the inhibition of energy-storage processes such as fatty acid biosynthesis. miR-122 inhibition can enhance p-AMPK activation and promote a shift in energy utilization through inhibition of ACC2 and FAS [10].

Synthetic drug categories, mostly effective as hypolipemics, are many; however, most of them exhibit variable side effects such as diarrhea, nausea, myositis, and impaired liver function. Natural products or traditional drugs are becoming more and more popular as alternative treatment options because of their safety [11].

10-Dehydrogingerdione (10-DHG), a biologically active compound derived from the plant ginger rhizome (*Zingiber officinale*), demonstrated potent anti-inflammatory, antioxidant, and hypolipemic characteristics and, additionally, remarkably increased nitric oxide release [12], and it proved to prevent the aortic calcifications in dyslipidemic rabbits [13].

Caffeic acid (CA) is a phenolic compound belonging to hydroxyl cinnamic acid derivatives, and it is available in human diets in berries, kiwis, plums, apples, many vegetables, and coffee [14]. A previous study indicated that CA and its derivatives have antibacterial, hypoglycemic, antioxidant, anti-inflammatory, anticancer, and cardiovascular protective effects [15]. Another study demonstrated its inhibitory effect on oxidative modification of LDL, mostly implicated in the development of atherosclerosis [16].

Accordingly, the present study aimed mainly to investigate the lipid-lowering effect of 10-DHG and CA, individually through targeting specific pathways, effective enzymes dealing with either lipogenesis, or lipolysis, focusing on the role of miR-122 and ACLY activity. A histological study of liver tissue was also done to demonstrate any correlation between the biomarkers studied and the histopathological findings.

## 2. Materials and Methods

### 2.1. Drugs and Chemicals

The 10-DHG was isolated from fresh rhizomes (*Zingiber officinale*), then identified and purified in the phytochemistry research lab, as shown previously [17]. CA and streptozotocin (STZ) were obtained from Sigma-Aldrich, Inc., St. Louis, MO, USA (C0625 and S0130, respectively). Cholesterol, cholic acid, and thiouracil were obtained from Loba chemie Pvt. Ltd., Colaba, Mumbai, India (02781, 02790, and 06286, respectively).

### 2.2. Animals

Twenty-four male albino rats (6–8 weeks old), 140–155 g body weight, were supplied from the faculty of veterinary medicine, Zagazig University, Egypt. The rats were acclimated for one week in the animal house of Zagazig University’s faculty of pharmacy under standard environmental conditions of 21–23 °C and a 12 h light–dark cycle, with free access to tap water and food. All the experimental procedures followed the National Institutes of Health (NIH) guidelines for animal handling and were approved by Zagazig University’s Institutional Animal Care and Use Committee (ZU-IACUC), permission number (3/F/119/2020).

### 2.3. Experimental Design

The rats were overnight fasted and divided into three groups (n = 6 each group) and received STZ solution (50 mg/kg) dissolved in citrate buffer (0.1 M, pH = 4.5, freshly prepared IP) [18]. To avoid hypoglycemic shock, 10% glucose was added to the drinking water for 24 h after STZ. The Bionime Rightest Wiz Plus^®^ glucometer (Bionime GmbH, Berneck, Switzerland) was used to check blood glucose levels after 72 h. Rats that achieved a blood glucose level of ≥250 mg/dl were expressed as diabetic [19]. Diabetic rats were fed CCT-diet (normal diet supplemented with 4% cholesterol and 1% cholic acid, along with 0.5% thiouracil in the drinking water) for two weeks [20,21]. One group continued for 6 weeks without any treatment, referred to as positive (+ve) control group, while the other two groups received 10-DHG (10 mg/kg/day) [22] or CA (50 mg/kg/day) [23] orally and individually for 6 weeks. Another separate group receiving a normal diet was included and referred to as the negative (-ve) control group.

### 2.4. Blood Sampling and Tissue Collection

After 6 weeks, the rats were fasted overnight; blood was drawn from the orbital sinus and allowed to coagulate for 15 min, then centrifuged for 15 min at 4000 rpm. Serum was collected and divided into two parts: one was instantly forwarded for glucose and lipid profile determinations, while the other was kept at −20 °C for any other additional biochemical analysis. Rats were later sacrificed by decapitation under deep anesthesia using diethyl ether (Merck), and the liver samples were isolated, rinsed in cold saline, dried, and then divided into several parts; one was directed for the evaluation of hepatic miR-122 and ACLY, p-AMPK, HMG-CoA reductase, and FAS activities. Another segment was quickly frozen in liquid nitrogen and kept at −20 °C. The remaining portion was kept at 4 °C for 72 h in 10% neutral buffered formalin before being processed for histological studies.

### 2.5. Analytical Procedures

Serum lipids (TC, TG, and HDL-C) and fasting blood glucose were determined using commercially available kits (#1001091, #1001311, #1001095, and #1001200, Spinreact Co., Sant Esteve de Bas, Girona, Spain, respectively). The Friedewald formula was used to determine LDL-C [24]. The atherogenic index was calculated using the LDL-C/HDL-C ratio (AI) [25]. Hepatic ACLY activity was evaluated using an ELISA kit obtained from (#E2908Ra, Jiaxing Korain Biotech Co. Ltd., Jiaxing, Zhejiang, China). Hepatic p-AMPK, HMG-CoA reductase, and FAS activities were determined using ELISA kits obtained from (#MBS765897, #MBS761708, and #MBS043636, My BioSource Inc., San Diego, CA, USA), respectively.

### 2.6. Quantitative Real Time PCR

The total RNA was first collected using the Trizol reagent (#15596026, Life Technologies Corporation, Carlsbad, CA, USA), then 1 μg of total RNA was reverse transcribed into cDNA by using a QuantiTects Reverse Transcription Kit (#205311, Qiagen Sciences Inc., Germantown, MD, USA). C-DNA was amplified via a Maximas SYBR Green/Fluorescein qPCR Master Mix (#K0241, Thermo Fisher Scientific Inc., Carlsbad, CA, USA) through specific primers that were prepared according to the manufacturer’s protocol (Table 1). For PCR assay, 12.5 μL Maxima SYBR Green/ Fluorescein qPCR Master Mix (2X) was mixed with 1 μL cDNA template, 0.3 μL forward primer, 0.3 μL reverse primer, and nuclease-free water to complete the volume to 25 μL. The conditions were designed as follows: 10 min at 95 °C, followed by 45 cycles of 95 °C for 10 s, 60 °C for 15 s, and 72 °C for 15 s. β-Actin was applied as an internal reference for miRNA. Rotor-Gene^®^ Q with software version 2.1.0 (Qiagen Sciences Inc., Germantown, MD, USA) collected data automatically and analyzed the value of the threshold cycle (Ct), which normalized to an average Ct value of the house-keeping genes (∆Ct); 2^-ΔΔCt^ was used for calculating relative gene expression fold [26].

### 2.7. Histological Examination

The liver samples fixed in 10% buffered neutral formalin solution for 48 h were dehydrated through ethanol upgrading from 75 to 100%, then cleaned with xylene and embedded in paraffin. Sections of liver were cut to a thickness of 3–5 µm and stained with hematoxylin and eosin (H&E) for histological study [27]. A lesions score system was evaluated as the following: (0 = no detectable histopathological alterations, 1 = rarely minimal or focal, 2 = multifocal, 3 = patchy or diffuse) with a semiquantitative method [28].

### 2.8. Statistical Analysis

The results were statistically analyzed using Prism 9 from GraphPad (San Diego, CA, USA) and presented as mean ± standard deviation (SD). One- and two-way ANOVA was used to assess significant differences between groups; both of them were followed by Tukey’s post hoc test for intergroup comparison. The significance level was set at *p* < 0.05.

## 3. Results

### 3.1. Effect of Caffeic Acid and 10-Dehydrogengerdione on Body Weight and Liver Weight

Table 2 shows the decreased body weight of the positive control rats compared with the negative control (*p* < 0.05). The 10-DHG and CA groups significantly gained weight compared with the positive control. Liver weight of the positive control rats demonstrated a significant increase compared with the negative control rats (*p* < 0.05) and turned out to be reduced by both drugs compared with the positive control rats (*p* < 0.05).

### 3.2. Effect of Caffeic Acid and 10-Dehydrogengerdione Treatment on miR-122 Expression

**Figure 1:**
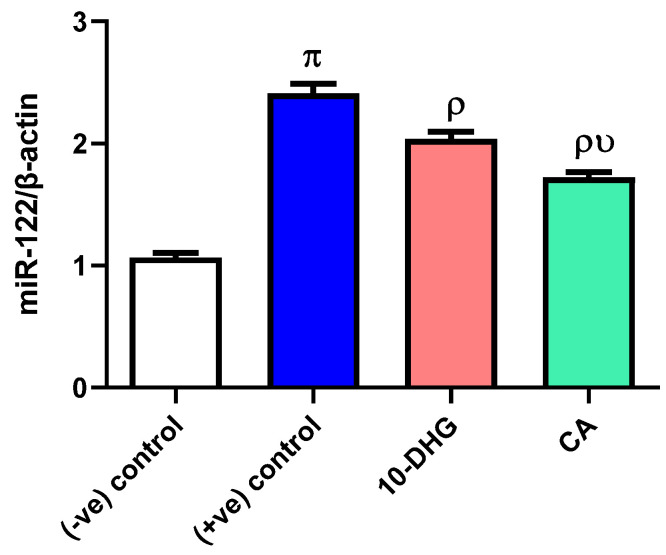
Effects of caffeic acid and 10-dehydrogengerdione treatment on miR-122 expression. (-ve) control: rats received standard rodent diet; (+ve) control: diabetic rats received CCT diet without receiving any treatment; 10-DHG: diabetic rats received CCT diet and 10-dehydrogengerdione (10 mg/kg/day); CA: diabetic rats received CCT diet and caffeic acid (50 mg/kg/day). Data are presented as mean ± (SD), n = 6 for each group; π *p* < 0.05 vs. (-ve) control, ρ *p* < 0.05 vs. (+ve) control, υ *p* < 0.05 vs. 10-DHG. Used test: one-way ANOVA.

Figure 1 demonstrates that diabetic hyperlipidemic rats showed a significant increase in miR-122 gene expression (2.3-fold) as compared to the negative control rats (*p* < 0.05). Administration of either 10-DHG or CA remarkably decreased miR-122 expression compared with the positive control (15.5% and 28.6%, respectively) (*p* < 0.05). Suppression of miR-122 expression by CA was remarkably more, as compared to 10-DHG (15.5%) (*p* < 0.05).

### 3.3. Effect of Caffeic Acid and 10-Dehydrogengerdione Treatment on Hepatic p-AMPK and Blood Glucose

**Figure 2:**
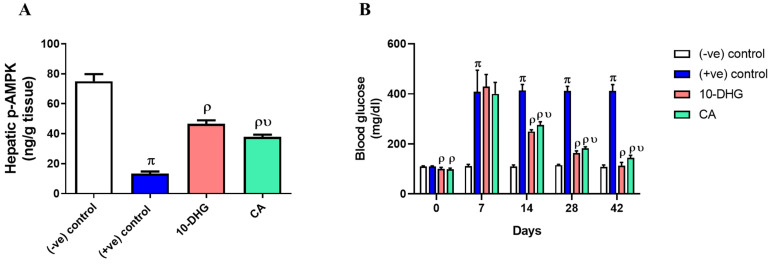
Effect of caffeic acid and 10-dehydrogengerdione treatment on (**A**) hepatic p-AMPK; (**B**) blood glucose. (-ve) control: rats received standard rodent diet; (+ve) control: diabetic rats received CCT diet without receiving any treatment; 10-DHG: diabetic rats received CCT diet and 10-dehydrogengerdione (10 mg/kg/day); CA: diabetic rats received CCT diet and caffeic acid (50 mg/kg/day). p-AMPK: phosphorylated form of AMP activated protein kinase. Data are presented as mean ± (SD), n = 6 for each group; π *p* <0.05 (-ve) control, ρ *p* < 0.05 vs. (+ve) control, υ *p* < 0.05 vs. 10-DHG. Used test: (**A**) one-way ANOVA; (**B**) two-way ANOVA.

The positive control group showed decreased p-AMPK activity as compared to the negative one (Figure 2A); 10-DHG and CA significantly increased p-AMPK activity (*p* < 0.05) compared with the positive control. The 10-DHG exhibited more enhancing effect (3.5-fold) compared with CA (2.9-fold).

Figure 2B illustrates that blood glucose levels in positive control rats demonstrated a significant increase compared with negative control rats and turned out to be decreased by 10-DHG and CA treatment.

### 3.4. Effect of Caffeic Acid and 10-Dehydrogengerdione Treatment on Hepatic ACLY, FAS, and Serum Triglyceride

**Figure 3:**
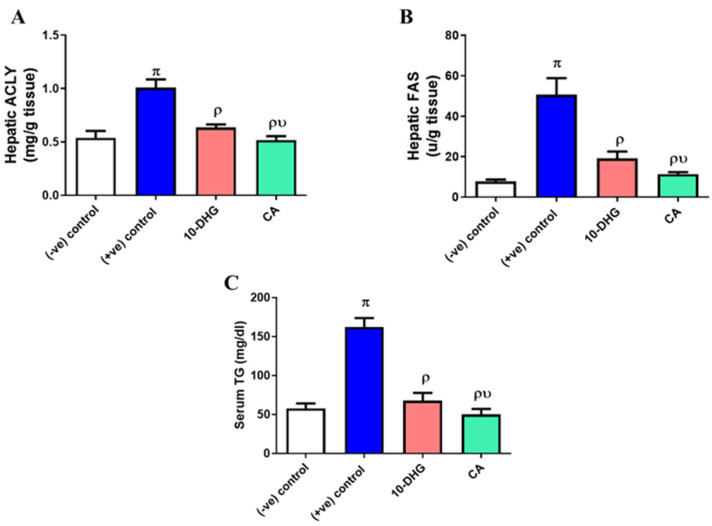
Effect of caffeic acid and 10-dehydrogengerdione treatment on hepatic (**A**) ACLY; (**B**) FAS and (**C**) serum triglyceride. (-ve) control: rats received standard rodent diet; (+ve) control: diabetic rats received CCT diet without receiving any treatment; 10-DHG: diabetic rats received CCT diet and 10-dehydrogengerdione (10 mg/kg/day); CA: diabetic rats received CCT diet and caffeic acid (50 mg/kg/day). ACLY: ATP citrate lyase; FAS: fatty acid synthase; TG: triglyceride; data are presented as mean ± (SD), n = 6 for each group; π *p* < 0.05 vs. (-ve) control; ρ *p* < 0.05 vs. (+ve) control, υ *p* < 0.05 vs. 10-DHG. Used test: one-way ANOVA.

In Figure 3A, the positive control group demonstrated significant elevation in ACLY activity compared with the negative control group (*p* < 0.05) and turned out to be reduced by treatment with 10-DHG and by CA. Furthermore, CA exhibited a substantial inhibitory effect on ACLY (18.4%) compared with 10-DHG (*p* < 0.05).

Moreover, the positive control group demonstrated increased activity of FAS (6.5-fold) compared with the negative control group (Figure 3B), dramatically reduced after treatment with CA (77.7%), and was more remarkable than 10-DHG treatment (62.1%) (*p* < 0.05).

Figure 3C illustrates that positive control rats demonstrated hypertriglyceridemia compared with the negative control ones (*p* < 0.05); 10-DHG or CA treatments significantly decreased TG levels (*p* < 0.05) compared with the positive control and were more remarkable than 10-DHG (*p* < 0.05).

### 3.5. Effect of Caffeic Acid and 10-Dehydrogengerdione Treatment on Hepatic HMG-CoA Reductase Activity and Lipogram Pattern

**Figure 4:**
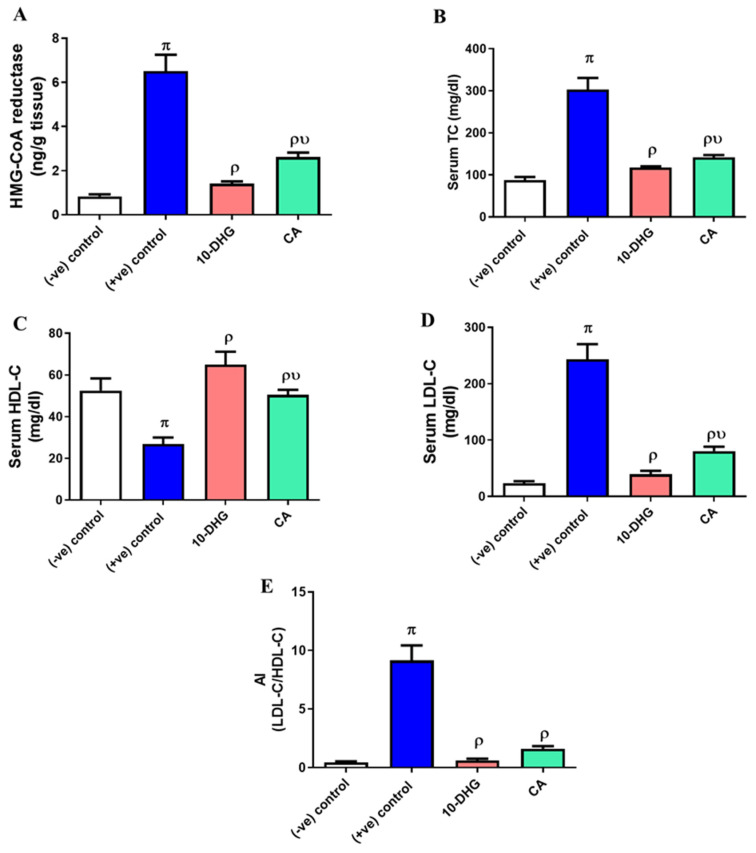
Effect of caffeic acid and 10-dehydrogengerdione treatment on (**A**) hepatic of HMG-CoA reductase; (**B**) serum TC; (**C**) serum HDL-C; (**D**) serum LDL-C and (**E**) AI. (-ve) control: rats received standard rodent diet; (+ve) control: diabetic rats received CCT diet without receiving any treatment; 10-DHG: diabetic rats received CCT diet and 10-dehydrogengerdione (10 mg/kg/day); CA: diabetic rats received CCT diet and caffeic acid (50 mg/kg/day). HMG-CoA: hydroxy methyl glutaryl coenzyme A; TC: total cholesterol; HDL-C: high density lipoprotein cholesterol; LDL-C: low density lipoprotein cholesterol; AI: atherogenic index. Data are presented as mean ± (SD), n = 6 for each group. π *p* < 0.05 vs. (-ve) control, ρ *p* < 0.05 vs. (+ve) control; υ *p* < 0.05 vs. 10-DHG. Used test: one-way ANOVA.

Figure 4A demonstrates that intake of either 10-DHG or CA significantly decreased HMG-CoA reductase activity by 78.3% and 59.7%, respectively, as compared to the positive control group, and was more remarkable in 10-DHG as compared to CA (*p* < 0.05).

As shown in Figure 4(B–E), 10-DHG or CA administration significantly decreased TC, LDL-C, and AI levels along with significant increase in HDL-C levels compared with positive controls (*p* < 0.05); 10-DHG achieved greater potential than CA regarding such lipids.

### 3.6. Effect of Caffeic Acid and 10-Dehydrogengerdione Treatment on Hepatic HMG-CoA Reductase Activity and Lipogram Pattern

**Figure 5:**
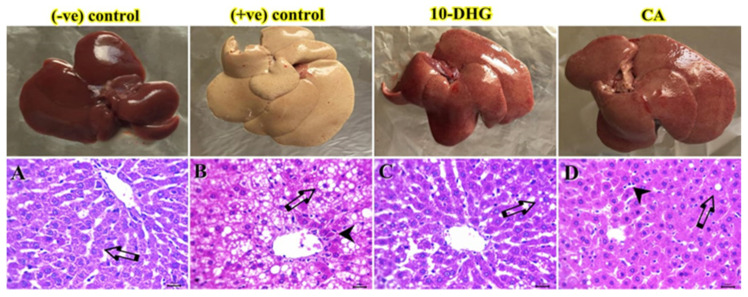
Effect of caffeic acid and 10-dehydrogengerdione on liver histology and morphology. Photomicrograph of H&E-stained sections from liver (scale bar 20 μm) showing: (**A**) normal architectures of hepatocytes (arrow), central vein and sinusoids in (-ve) control group; (**B**) intense lipidosis beside presence of foamy cells with centrally located nuclei (arrow) and apoptotic cells (arrowhead) in (+ve) control group; (**C**) unicellular hepatic lipidosis (arrow) in CA; (**D**) fatty change in individual cell (arrow), as well as lymphocytic infiltrates within some sinusoids (arrowhead) in 10-DHG.

Hepatocytes of the negative control group exhibited normal patterns of architecture, central veins, sinusoids, and portal triads. Diabetic hyperlipidemic rats revealed intense centrilobular and periportal lipidosis in (50%) of examined sections. The majority of the degenerated hepatic cells were foamy with centrally located nuclei. Furthermore, minute foci of necrosis, apoptotic cell, and periportal inflammatory cell aggregates (mainly lymphocytes) were commonly observed lesions. Unicellular hepatic lipidosis was noticed in 10% of hepatic parenchyma in the CA group, while the 10-DHG group showed hepatic parenchyma, mostly ameliorated along with fatty change in individualized hepatocytes and, additionally, lymphocytic infiltrates within some sinusoids were also detected (Figure 5).

Table 3 shows a summary of a semiquantitative lesion score system for the liver alteration, in which diabetic hyperlipidemic rats demonstrated a significant alteration in hepatic parenchyma as lipidosis with multiple foamy-appearing cells, necrotic, apoptotic, and lymphocytes infiltrates as compared to the negative (-ve) control rats. Rats treated with 10-DHG induced more amelioration of hepatic histological lesion scores than those treated with CA.

## 4. Discussion

The present study illustrates the hypolipidemic and hypoglycemic effects of CA and 10-DHG in diabetic hyperlipidemic rats. This was attributed mostly to inhibition of miR-122 and liver lipogenic enzymes (FAS and ACLY), along with p-AMPK activation.

miR-122, as reported before, can regulate fat metabolism in the liver, meaning that its overexpression or suppression may induce changes in the synthesis of fatty acids and cholesterol. It was indicated also that using antisense oligonucleotides to block miR-122 expression in normal mice significantly decreased cholesterol levels, hepatic fatty acid, and cholesterol synthesis and, additionally, activation of fatty acid oxidation and p-AMPK. This can block the activity of important enzymes involved in the synthesis of cholesterol and fatty acids. Blocking of miR-122 expression in obese mice models using the same technique resulted in repression of plasma cholesterol and improved liver tissue steatosis by repressing the gene expressions related to fatty acid synthesis such as FAS and ACLY [10]. miR-122 inhibition also significantly decreased plasma cholesterol through repression of genes involved in cholesterol synthesis such as HMG-CoA reductase and HMG-CoA synthase [6].

The present study, in agreement, demonstrated similar findings to those mentioned above. Taking into consideration that the metabolic energy balance in the entire body is controlled by p-AMPK, a critical cellular energy sensor Liver p-AMPK activation stimulates, in turn, certain catabolic pathways, resulting in hypoglycemic and lipolytic effects [29] and concomitant suppression of miR-122 in the liver activates hepatic p-AMPK [30]. This may be mediated either directly or indirectly where p-AMPK activities showed an inverse correlation with miR-122 expression [31]. Accordingly, increased activity of hepatic p-AMPK by CA and 10-DHG intake might be attributed to inhibition of MiR-122 expression in our diabetic hyperlipidemic rat model.

FAS is an essential metabolic enzyme that catalyzes the conversion of acetyl CoA and monoacyl malonate CoA into palmitic acid [32]. A previous study reported that miR-122 regulated FAS in an indirect manner via an unidentified pathway [33]. The present results were in agreement with these findings; meanwhile, the expression of some lipogenic genes as FAS showed a dramatic increase following the overexpression of miR-122 in HFD rats, leading to lipid accumulation [1,34,35].

HMG-CoA reductase represents the rate-limiting step in cholesterol biosynthesis where it catalyzes the conversion of HMG-CoA into the mevalonate step [36]. Many reports illustrated that miR-122 suppression induced a reduction in HMG CoA reductase expression and, indeed, a plasma cholesterol decrease [10,37]. Administration of 10-DHG in the present study significantly decreased plasma cholesterol and might be attributed to reductions in both miR-122 expression and HMG-CoA reductase activity.

It had been reported before that certain phenolic compounds (plant derived) significantly downregulated liver miR-122 expression in mice [38,39].

CA is a phenolic compound (plant derived) and is widely present in nature, and its potential to increase p-AMPK activation and to decrease FAS and HMG-CoA reductase activities was reported before [40]. Another study in human-cell carcinoma lines reported that CA had an inhibitory effect on the expression of FAS and ACLY [41]. A recent study indicated also that daily administration of CA for 5 weeks resulted in significant hypoglycemia in diabetic rats, joined with a remarkable antioxidant effect [42]. The present study was in agreement with these findings and might explain the hypolipemic effect of CA.

A previous study demonstrated ginger extract’s potential to suppress hepatic de novo lipogenesis and attributed it to a hepatic mRNA-level decrease in lipogenic enzymes such as FAS [43]. It also prevented the HFD-induced elevation of HMG-CoA reductase protein in the rat liver, affecting, in turn, cholesterol biosynthesis [44], and additionally decreased significantly the blood sugar levels in diabetic animal models [45,46]. Accordingly, the reduction observed in hepatic FAS, ACLY, and HMG-CoA reductase along with hepatic p-AMPK activation leading to decreased TC and TG along with blood glucose improvement after 10-DHG intake may present an explanation for its mechanism as a hypolipemic agent. CA intake induced in turn and to certain extent similar findings as compared to the 10-DHG results.

## Figures and Tables

**Table 1:** Primers sequence for RT-PCR.

Gene	Sequence
**miR-122**	F: 5′-GGGGTGGAGTGTGACAATG-3
	R: 5′-CAGTGCGTGTCGTGGAGT-3′
**β-Actin**	F: 5′-GCCGGGACCTGACTGACTAC-3
	R: 5′-TTCTCCTTAATGTCACGCACGAT-3

**Table 2:** Effect of caffeic acid and 10-dehydrogengerdione on body weight and liver weight.

	Parameters	Initial Body Weight	Final Body Weight	Liver Weight	Liver Weight/Final Body Weight Ratio (%)
Groups					
(-ve) control		146.2 ± 2.64	337.2 ± 6.76	8.29 ± 0.53	2.56 ± 0.21
(+ve) control		151 ± 6.60	174.8 ± 7.33 π	16.10 ± 0.44 π	9.22 ± 0.49 π
10-DHG		149.3 ± 5.05	292.2 ± 37.79 ρ	11.32 ± 1.72 ρ	3.9 ± 0.69 ρ
CA		147.4 ± 4.23	288 ± 16.19 ρ	11.17 ± 0.25 ρ	3.86 ± 0.25 ρ

(-ve) control: rats received standard rodent diet; (+ve) control: diabetic rats received CCT diet without receiving any treatment; 10-DHG: diabetic rats received CCT diet and 10-dehydrogengerdione (10 mg/kg/day); CA: diabetic rats received CCT diet and caffeic acid (50 mg/kg/day). Data are presented as mean ± (SD), n = 6 for each group; π *p* < 0.05 vs. (-ve) control, ρ *p* < 0.05 vs. (+ve) control. Used test: one-way ANOVA.

**Table 3:** Lesion scores of the severity extent in the hepatic tissues among different groups.

Organ	Main Lesions	(-ve) Control	(+ve) Control	10-DHG	CA
Liver	Acute cell swelling	0	2	0	1
	Lipidosis and foamy cells	0	3	1	1
	Necrotic and apoptotic cells	0	2	0	1
	Lymphocytes infiltrates	0	2	1	1

(-ve) control: rats received standard rodent diet; (+ve) control: diabetic rats received CCT diet without receiving any treatment; 10-DHG: diabetic rats received CCT diet and 10-dehydrogengerdione (10 mg/kg/day); CA: diabetic rats received CCT diet and caffeic acid (50 mg/kg/day). The represented scores in the table are the mean lesion scores. Histological lesions were scored for severity as follows (0 = no detectable histopathological lesion, 1 = rarely minimal or focal, 2 = multifocal, 3 = patchy or diffuse) as a semiquantitative method.

## Data Availability

The datasets generated during and/or analyzed during the current study are available from the corresponding author upon reasonable request.

## References

[1] Su D., Zhang R., Hou F., Chi J., Huang F., Yan S., Liu L., Deng Y., Wei Z., Zhang M. (2017). Lychee pulp phenolics ameliorate hepatic lipid accumulation by reducing miR-33 and miR-122 expression in mice fed a high-fat diet. Food Funct..

[2] Baselga-Escudero L., Pascual-Serrano A., Ribas-Latre A., Casanova E., Salvadó M.J., Arola L., Arola-Arnal A., Bladé C. (2015). Long-term supplementation with a low dose of proanthocyanidins normalized liver miR-33a and miR-122 levels in high-fat diet-induced obese rats. Nutr. Res..

[3] Rottiers V., Näär A.M. (2012). MicroRNAs in metabolism and metabolic disorders. Nat. Rev. Mol. Cell Biol..

[4] Hsu S.H., Wang B., Kota J., Yu J., Costinean S., Kutay H., Yu L., Bai S., La Perle K., Chivukula R.R. (2012). Essential metabolic, anti-inflammatory, and anti-tumorigenic functions of miR-122 in liver. J. Clin. Investig..

[5] Baselga-Escudero L., Bladé C., Ribas-Latre A., Casanova E., Salvadó M.J., Arola L., Arola-Arnal A. (2012). Grape seed proanthocyanidins repress the hepatic lipid regulators miR-33 and miR-122 in rats. Mol. Nutr. Food Res..

[6] Rotllan N., Fernández-Hernando C. (2012). MicroRNA Regulation of Cholesterol Metabolism. Cholesterol.

[7] Rahayu Lestari S., Lukiati B., Nur Arifah S., Rofiqotun Nurul Alimah A., Gofur A. (2019). Medicinal Uses of Single Garlic in Hyperlipidemia by Fatty Acid Synthase Enzyme Inhibitory: Molecular Docking. IOP Conf. Ser. Earth Environ. Sci..

[8] Pinkosky S.L., Groot P.H.E., Lalwani N.D., Steinberg G.R. (2017). Targeting ATP-Citrate Lyase in Hyperlipidemia and Metabolic Disorders. Trends Mol. Med..

[9] Xie Z., Zhang M., Song Q., Cheng L., Zhang X., Song G., Sun X., Gu M., Zhou C., Zhang Y. (2022). Development of the novel ACLY inhibitor 326E as a promising treatment for hypercholesterolemia. Acta Pharm. Sin. B.

[10] Esau C., Davis S., Murray S.F., Yu X.X., Pandey S.K., Pear M., Watts L., Booten S.L., Graham M., McKay R. (2006). miR-122 regulation of lipid metabolism revealed by in vivo antisense targeting. Cell Metab..

[11] Li Z.Y., Ding L.L., Li J.M., Xu B.L., Yang L., Bi K.S., Wang Z.T. (2015). ¹H-NMR and MS based metabolomics study of the intervention effect of curcumin on hyperlipidemia mice induced by high-fat diet. PLoS ONE.

[12] Chao C.-T., Yeh H.-Y., Tsai Y.-T., Chuang P.-H., Yuan T.-H., Huang J.-W., Chen H.-W. (2019). Natural and non-natural antioxidative compounds: Potential candidates for treatment of vascular calcification. Cell Death Discov..

[13] Elseweidy M.M., Mohamed H.E., Elrashidy R.A., Atteia H.H., Elnagar G.M., Ali A.E.-M. (2019). Potential therapeutic roles of 10-dehydrogingerdione and/or pentoxifylline against calcium deposition in aortic tissues of high dietary cholesterol-fed rabbits. Mol. Cell. Biochem..

[14] Silva H., Lopes N.M.F. (2020). Cardiovascular Effects of Caffeic Acid and Its Derivatives: A Comprehensive Review. Front. Physiol..

[15] Ekeuku S.O., Pang K.L., Chin K.Y. (2021). Effects of Caffeic Acid and Its Derivatives on Bone: A Systematic Review. Drug Des. Dev. Ther..

[16] Khan F.A., Maalik A., Murtaza G. (2016). Inhibitory mechanism against oxidative stress of caffeic acid. J. Food Drug Anal..

[17] Choi S.Y., Park G.S., Lee S.Y., Kim J.Y., Kim Y.K. (2011). The conformation and CETP inhibitory activity of [10]-dehydrogingerdione isolated from *Zingiber officinale*. Arch. Pharm. Res..

[18] Tripathi A.K., Gupta P.S., Singh S.K. (2019). Antidiabetic, anti-hyperlipidemic and antioxidant activities of Bauhinia variegata flower extract. Biocatal. Agric. Biotechnol..

[19] Jaishree V., Narsimha S. (2020). Swertiamarin and quercetin combination ameliorates hyperglycemia, hyperlipidemia and oxidative stress in streptozotocin-induced type 2 diabetes mellitus in wistar rats. Biomed. Pharmacother. Biomed. Pharmacother..

[20] Elseweidy M.M., Elnagar G.M., Elsawy M.M., Ali A.A., Zein N. (2020). Losartan and azelastine either alone or in combination as modulators for endothelial dysfunction and platelets activation in diabetic hyperlipidemic rats. J. Pharm. Pharmacol..

[21] Sethi A., Parmar H.S., Kumar A. (2011). The effect of aspirin on atherogenic diet-induced diabetes mellitus. Basic Clin. Pharmacol. Toxicol..

[22] Elseweidy M.M., Elnagar G.M., Elsawy M.M., Zein N. (2022). Azelastine a potent antihistamine agent, as hypolipidemic and modulator for aortic calcification in diabetic hyperlipidemic rats model. Arch. Physiol. Biochem..

[23] Wang Y., Kaur G., Kumar M., Kushwah A.S., Kabra A., Kainth R. (2022). Caffeic Acid Prevents Vascular Oxidative Stress and Atherosclerosis against Atherosclerogenic Diet in Rats. Evid.-Based Complement. Altern. Med..

[24] Friedewald W.T., Levy R.I., Fredrickson D.S. (1972). Estimation of the concentration of low-density lipoprotein cholesterol in plasma, without use of the preparative ultracentrifuge. Clin. Chem..

[25] Sharma H., Pathan R.A., Kumar V., Javed S., Bhandari U. (2011). Anti-apoptotic potential of rosuvastatin pretreatment in murine model of cardiomyopathy. Int. J. Cardiol..

[26] Schmittgen T.D., Livak K.J. (2008). Analyzing real-time PCR data by the comparative C(T) method. Nat. Protoc..

[27] Suvarna K.S., Layton C., Bancroft J.D. (2018). Bancroft’s Theory and Practice of Histological Techniques E-Book.

[28] Gibson-Corley K.N., Olivier A.K., Meyerholz D.K. (2013). Principles for valid histopathologic scoring in research. Vet. Pathol..

[29] Hardie D.G., Ross F.A., Hawley S.A. (2012). AMPK: A nutrient and energy sensor that maintains energy homeostasis. Nat. Rev. Mol. Cell Biol..

[30] Shizu R., Shindo S., Yoshida T., Numazawa S. (2012). MicroRNA-122 down-regulation is involved in phenobarbital-mediated activation of the constitutive androstane receptor. PLoS ONE.

[31] Kwon I.G., Ha T.K., Ryu S.-W., Ha E. (2015). Roux-en-Y gastric bypass stimulates hypothalamic miR-122 and inhibits cardiac and hepatic miR-122 expressions. J. Surg. Res..

[32] Zhang W., Li J.Y., Wei X.C., Wang Q., Yang J.Y., Hou H., Du Z.W., Wu X.A. (2021). Effects of dibutyl phthalate on lipid metabolism in liver and hepatocytes based on PPARα/SREBP-1c/FAS/GPAT/AMPK signal pathway. Food Chem. Toxicol..

[33] Liu S., Wu Z., Guo S., Meng X., Chang X. (2018). Polyphenol-rich extract from wild Lonicera caerulea berry reduces cholesterol accumulation by mediating the expression of hepatic miR-33 and miR-122, HMGCR, and CYP7A1 in rats. J. Funct. Foods.

[34] Baselga-Escudero L., Arola-Arnal A., Pascual-Serrano A., Ribas-Latre A., Casanova E., Salvadó M.J., Arola L., Blade C. (2013). Chronic administration of proanthocyanidins or docosahexaenoic acid reverses the increase of miR-33a and miR-122 in dyslipidemic obese rats. PLoS ONE.

[35] Liu L., Zhao J., Li Y., Wan Y., Lin J., Shen A., Xu W., Li H., Zhang Y., Xu J. (2016). Artemisia capillaris formula inhibits hepatic steatosis via an miR-122-induced decrease in fatty acid synthase expression in vivo and in vitro. Mol. Med. Rep..

[36] Friesen J.A., Rodwell V.W. (2004). The 3-hydroxy-3-methylglutaryl coenzyme-A (HMG-CoA) reductases. Genome Biol..

[37] Krützfeldt J., Rajewsky N., Braich R., Rajeev K.G., Tuschl T., Manoharan M., Stoffel M. (2005). Silencing of microRNAs in vivo with ‘antagomirs’. Nature.

[38] de Alencar Silva A., Pereira-de-Morais L., Rodrigues da Silva R.E., de Menezes Dantas D., Brito Milfont C.G., Gomes M.F., Araújo I.M., Kerntopf M.R., Alencar de Menezes I.R., Barbosa R. (2020). Pharmacological screening of the phenolic compound caffeic acid using rat aorta, uterus and ileum smooth muscle. Chem. Biol. Interact..

[39] Joven J., Espinel E., Rull A., Aragonès G., Rodríguez-Gallego E., Camps J., Micol V., Herranz-López M., Menéndez J.A., Borrás I. (2012). Plant-derived polyphenols regulate expression of miRNA paralogs miR-103/107 and miR-122 and prevent diet-induced fatty liver disease in hyperlipidemic mice. Biochim. Biophys. Acta (BBA) Gen. Subj..

[40] Liao C.C., Ou T.T., Wu C.H., Wang C.J. (2013). Prevention of diet-induced hyperlipidemia and obesity by caffeic acid in C57BL/6 mice through regulation of hepatic lipogenesis gene expression. J. Agric. Food Chem..

[41] Tyszka-Czochara M., Bukowska-Strakova K., Kocemba-Pilarczyk K.A., Majka M. (2018). Caffeic Acid Targets AMPK Signaling and Regulates Tricarboxylic Acid Cycle Anaplerosis while Metformin Downregulates HIF-1α-Induced Glycolytic Enzymes in Human Cervical Squamous Cell Carcinoma Lines. Nutrients.

[42] Xu W., Luo Q., Wen X., Xiao M., Mei Q. (2020). Antioxidant and anti-diabetic effects of caffeic acid in a rat model of diabetes. Trop. J. Pharm. Res..

[43] Gao H., Guan T., Li C., Zuo G., Yamahara J., Wang J., Li Y. (2012). Treatment with ginger ameliorates fructose-induced Fatty liver and hypertriglyceridemia in rats: Modulation of the hepatic carbohydrate response element-binding protein-mediated pathway. Evid.-Based Complement. Altern. Med. Ecam.

[44] Roufogalis B.D. (2014). *Zingiber officinale* (Ginger): A Future Outlook on Its Potential in Prevention and Treatment of Diabetes and Prediabetic States. New J. Sci..

[45] Al Hroob A.M., Abukhalil M.H., Alghonmeen R.D., Mahmoud A.M. (2018). Ginger alleviates hyperglycemia-induced oxidative stress, inflammation and apoptosis and protects rats against diabetic nephropathy. Biomed. Pharmacother. Biomed. Pharmacother..

[46] Alshathly M.R. (2019). Efficacy of Ginger (*Zingiber officinale*) in Ameliorating Streptozotocin-Induced Diabetic Liver Injury in Rats: Histological and Biochemical Studies. J. Microsc. Ultrastruct..

